# Relation between Conventional and Starch-Assisted ASP Injection and Impact of Crystallinity on Flood Formation

**DOI:** 10.3390/molecules28186685

**Published:** 2023-09-18

**Authors:** Hasanain A. Al-Jaber, Agus Arsad, Sulalit Bandyopadhyay, Mohd Zaidi Jaafar, Muhammad Tahir, Mustafa Jawad Nuhma, Abdulmunem R. Abdulmunem, Mohammad Yasin Abdulfatah, Hajar Alias

**Affiliations:** 1 UTM-MPRC Institute for Oil and Gas, Faculty of Engineering, Universiti Teknologi Malaysia, UTM, Skudai 81310, Johor, Malaysia; 2 Department of Chemical Industries Technologies, Southern Technical University, Zubair, Basrah 61006, Iraq; 3 Department of Chemical Engineering, Norwegian University of Science and Technology, Høgskoleringen 1, 7491 Trondheim, Norway; 4 Department of Chemical Engineering, School of Chemical and Energy Engineering, Universiti Teknologi Malaysia, Johor Bahru 81310, Johor, Malaysia; 5 Chemical and Petroleum Engineering Department, United Arab Emirates University (UAEU), Al Ain P.O. Box 15551, United Arab Emirates; 6 Chemical Engineering Department, College of Engineering, University of Al-Qadisiyah, Al-Diwaniyah P.O. Box 88, Iraq; 7 Electromechanical Engineering Department, University of Technology-Iraq, Baghdad 10066, Iraq; 8 Exploration and Development Department, PT SPR Langgak, Jakarta 12550, Indonesia

**Keywords:** conventional ASP flooding, improved ASP formation, cassava starch nanoparticles, nanoparticles derived from purple yam, characterization of biopolymers

## Abstract

Alkaline–surfactant–polymer (ASP) flooding, a recognized method for oil recovery, encounters limited use due to its expense. In addition, ASP’s best composition and injection sequence still remains uncertain today. This study explores conventional ASP flooding using PT SPR Langgak’s special surfactants, simulating Langgak oilfield conditions in Sumatra, Indonesia. By comparing the outcomes of this flooding technique with that of starch-assisted ASP performed in another study, the benefits of adding starch nanoparticles to flooding are evident. Nano-starch ASP increased oil recovery by 18.37%, 10.76%, and 10.37% for the three configurations investigated in this study. Water flooding preceded ASP flooding, and flooding operations were carried out at 60 °C. This study employed sodium hydroxide (NaOH), sodium carbonate (Na_2_CO_3_), and specialized surfactants from PT SPR. The adopted polymer is solely hydrolyzed polyacrylamide (HPAM) at 2000 ppm. Starch nanoparticles underwent comprehensive characterization and focused more on charge stability. Purple yam nanoparticles (PYNPs) exhibited remarkable stability at −36.33 mV, unlike cassava starch nanoparticles (CSNPs’) at −10.68 mV and HPAM’s at −27.13 mV. Surface properties affect interactions with fluids and rocks. Crystallinity, a crucial characterization, was assessed using Origin software 2019b. CSNPs showed 24.15% crystallinity, surpassing PYNPs’ 20.68%. Higher crystallinity benefits CSNPs’ thermal stability. The amorphous behavior found in PYNPs makes them less suitable if applied in harsh reservoirs. This research correlated with prior findings, reinforcing starch nanoparticles’ role in enhancing oil recovery. In summary, this study highlighted conventional ASP flooding using HPAM as the sole polymer and compared it with three formations that used two starch nanoparticles included with HPAM, assessing their impact on charge stability, crystallinity, and recovery rate to emphasize their importance in the oil recovery industry. Starch nanoparticles’ benefits and limitations guided further investigation in this study.

## 1. Introduction

Chemical flooding, especially alkaline–surfactant–polymer (ASP), plays an important role in enhancing oil recovery because of the synergy of its three components: alkaline; surfactant; and polymer. It is a very promising technology that can make incremental oil recovery of up to 30% from the original oil in place (OOIP) [1]. Lowering the interfacial tension (IFT) between oil and water phases, changing the rock wettability from oil-wet to water-wet, emulsifying the oil and water, and improving the mobility ratio between the displaced solution and the displacing one are the four basic mechanisms that interpret the role of ASP flooding in improving oil recovery [2,3,4,5]. After injection of the ASP stream, the naphthenic acids, which are available in the crude oil, react with the alkaline component to produce what it is called an “in situ surfactant”. The generated surfactant is combined with already an available surfactant in the injection stream [6]. To reduce the IFT between the water and oil phases, it is crucial to ensure that an appropriate amount of surfactant is present in the ASP flood solution [7]. The presence of polymer in the ASP solution acts like a modifier. This modifier can increase the viscosity of the input stream and enhance the sweep efficiency [8].

Regarding the alkaline utilized in ASP formulations, according to certain studies, employing caustic soda (NaOH), which is a potent alkali, can highly increase oil recovery [9,10]. Whereas other studies declared that recovering oil was more efficient when using weak alkali like sodium carbonate (Na_2_CO_3_) [11,12,13]. Yin et al. [11] conducted a number of tests to find the best alkali and its practical concentration that would likely produce higher recovery. When they used Na_2_CO_3_ at a concentration of 1.2 wt.%, they noticed that IFT dropped to a low value of only 1.13 mN/m, which is considered favorable for higher oil recovery. Additionally, they found that the wettability of the core sample was effectively altered to become water-wet, which is also considered a beneficial outcome. Efforts to enhance the efficiency and cost-effectiveness of ASP flooding have faced many challenges since its application as a tertiary process. According to Arsad et al. [14], achieving a higher incremental recovery requires that the oil–water interfacial tension reach a level of 10^−3^ mN/m, which is not easily attainable by only selecting one kind and concentration of the applied surfactant. Arsad et al. [14] added increasing the alkali content can significantly reduce the IFT, but this can potentially lead to some issues, such as decreasing the viscosity of the ASP stream. To address this concern, adding additional polymer to compensate for the viscosity reduction is recommended. Furthermore, a high concentration of alkali may give rise to some technical scaling problems within the piping system and instruments. Hence, it is essential to carefully adjust the alkali concentration to mitigate these challenges and ensure optimal functioning. Currently, there are 32 ASP projects worldwide, with 19 of them specifically implemented in China [15]. Technically, up-to-date ASP flooding can result in oil recovery that is 10% higher than polymer flooding and 20% higher than water flooding [16]. The existing stockpiles available for ASP flooding reach over 8300 million tons worldwide, with the Daqing oil field in China holding the potential to extract more than 1400 million tons alone [17]. However, ASP flooding faces economic limitations due to the substantial volumes of chemicals required. Thus, it is essential to consider both the technical and economic feasibility of ASP flooding to ensure the effective utilization of injected chemicals.

Over the past few decades, surfactants and polymers have been extensively employed to enhance oil recovery. However, in recent times, nanoparticles (NPs) have garnered considerable interest owing to their distinctive characteristics. Numerous types of NPs have been studied for their potential to enhance oil recovery, either individually or in conjunction with surfactants and/or polymers. Keykhosravi et al. [18] investigated the viscosity of Xanthan gum polymer both with and without titanium dioxide (TiO_2_) NPs. The potential of nano-polymer suspension to alter the wettability of the rock sample was assessed through contact angle measurements supported by a mathematical approach. The findings indicated that increasing the temperature and salinity degree of the nano-polymeric solution led to a decrease in shear viscosity. The formation of a nano-polymer using a TiO_2_ NPs suspension with Xanthan polymer extended the alteration of wettability compared to polymer-free nanofluid. A mathematical model based on kinetic adsorption and was developed to describe the wettability alteration and to identify contact angle profiles. Results from oil displacement tests revealed that TiO_2_ nanofluid and polymer solution using Xanthan gum exhibited additional oil recovery of 12% and 19% of OOIP, respectively [18]. The application of TiO_2_-induced Xanthan gum flooding resulted in an impressive increase of 25% in oil production.

A new EOR technique was proposed by Wang et al. [19], which combines traditional weak alkaline Na_2_CO_3_ ASP flooding with nanotechnology to assess its effectiveness at reservoir temperature, resulting in IFT and wettability in addition to oil recovery. The experimental findings revealed that ASP plus silicon dioxide (SiO_2_) as an NP mixture displayed superior performance in reducing the IFT between oil and water as well as altering their contact angle in comparison to a solution consisting solely of hydrolyzed polyacrylamide (HPAM) or traditional ASP. Furthermore, ASP with a SiO_2_ NP mixture demonstrated better displacement efficiency after water flooding in comparison to an HPAM/traditional ASP solution. Notably, the addition of SiO_2_ nanoparticles to the ASP formulation led to an improvement in oil recovery by 6.67% from OOIP compared to traditional ASP [19].

In the current study, the performance of crystalline NPs made from cassava and purple yam starches with HPAM as a hybrid polymer in combination with ASP is investigated and compared with traditional ASP that contains only HPAM in its polymer counterpart. This modified procedure of chemical flooding is utilized to improve the recovery ratio of heavy oil brought from the Langgak oilfield in Sumatra, Indonesia. The results of such a chemical flooding are discussed in detail in research conducted by Al-Jaber, H.A. et al. [20]. The current study is based on the comparison of this improved ASP flooding with traditional ASP flooding (without NPs), from which the polymer counterpart consisted only of HPAM. Also, the crystallinity degree of cassava and purple yam NPs is evaluated using a graphical interface method based on the Origin application utility. The new bio-polymers in the ASP formation can lead to the recovery of excess oil from matured reservoirs. Additionally, this combined polymer flooding may reduce the operational cost because of the abundance of cassava and purple yam roots in nature.

## 2. Results and Discussion

### 2.1. Properties of Produced Nanoparticles

**Figure 1:**
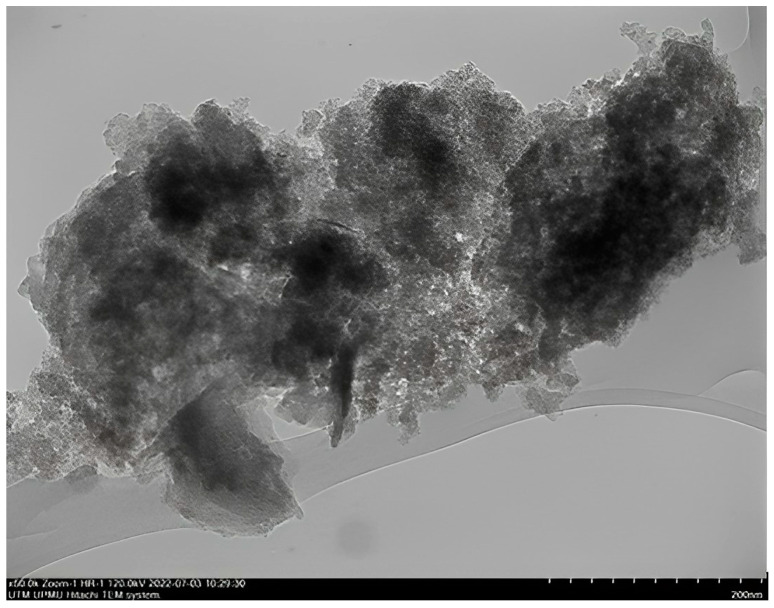
TEM image for CSNPs showing homogenous-sized particles.

**Figure 2:**
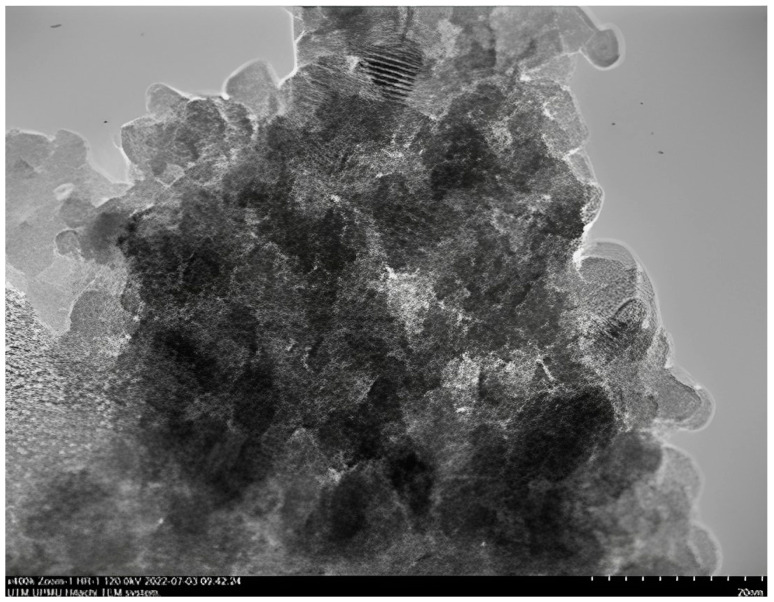
TEM image for PYNPs showing homogenous-sized particles.

This study demonstrates the potential of nanoparticles produced from CAS and PYS and the eco-friendly nature of the production method. Produced nanoparticles underwent precise analysis utilizing TEM through the instrument brand JEOL JEM-2100 TEM and FTIR analysis through a Bruker Vertex 70 FTIR spectrometer manufactured by Bruker Optics, Germany. These analyses are useful for unraveling nanoparticles’ morphological attributes, their particle size, and structural composition. The nanoparticles were synthesized using a sustainable approach [21], achieving notable yields of 90.53% for CSNPs and 85% for PYNPs. TEM images provided a captivating glimpse of nanoparticle structures, showcasing CSNPs with an average diameter of 52.92 nm. These nanoparticles exhibited mostly a uniform spherical morphology (Figure 1). In contrast, PYNPs displayed a larger average size of 363.12 nm (Figure 2). This discrepancy might be attributed to the inherent variations in starch tubers, leading to diverse nanoparticle characteristics [22].

As highlighted by Ku and Maynard [23], the amalgamation of unipolar electric influences acting on these nanoparticles, overseen by the temperature applied, has significantly contributed to the creation of a substantial proportion of dispersed and spherical nanoparticles. As a rapid reduction in particle interaction occurred due to sudden cooling and dilution influences [24], the particles underwent a transition from their initial spherical configuration to alternative shapes like hexagonal and rod-like structures. Moreover, the cavitation process may lead to the generation of free radicals, resulting from the creation, expansion, and collapse of bubbles among the nanoparticles [25].

**Figure 3:**
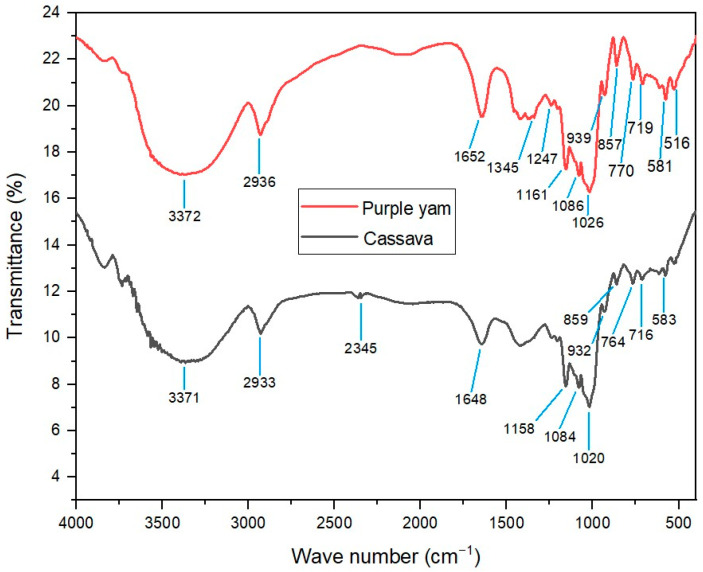
Interrelation of FTIR patterns for both CSNPs and PYNPs.

FTIR analysis provided valuable insights into the chemical composition and functional groups of both CSNPs and PYNPs. As seen in Figure 3, a set of close peaks was observed, indicating the presence of similar functional groups in both nanoparticles, while the additional peaks in the PYNPs spectrum suggest potential modification. The peak at 1020 cm^−1^ for CSNPs and 1026 cm^−1^ PYNPs corresponded to the stretching of S=O bonds that belong to sulfoxide compounds in linkages of starch molecules [26]. Interestingly, the peak of 1652 cm^−1^ for PYNPs could be attributed to the presence of carbonyl groups, possibly indicating the occurrence of oxidative reactions during the nanoparticle synthesis process [27]. This suggests that PYNPs might have undergone some degree of chemical modification during their formation. Comparing the FTIR spectra of CSNPs and PYNPs, it is evident that both nanoparticles share a close structural framework, with variations primarily in terms of additional peaks in the PYNPs spectrum. These variations suggest possible differences in bonding interactions and the chemical composition between the two types of nanoparticles.

### 2.2. Surface Charge and Stability of Nanoparticles

In this study, we investigated the zeta potential for the two distinct nanoparticle types: CSNPs and PYNPs, to obtain a complete picture of the stability of these produced particles. Our analysis encompassed three independent runs for each nanoparticle type, allowing for a comprehensive comparison of their zeta potential. Their values are compared with that of an HPAM solution to investigate further the improvement achieved by using these particles in the injection stream. The evaluation process was carried out at URIL, UTM University.

For CSNPs, zeta potential values recorded for three successive runs were found to be −9.3, −10.3, and −12.4 mV. Therefore, the average value for this potential yielded was a value of −10.68 mV. On the other hand, PYNPs exhibited zeta potential values of −43.9, −33.8, and −31.3 mV across these runs. The resulting average zeta potential for PYNPs was −36.33 mV. These findings indicate that PYNPs possess significantly higher stability compared to CSNPs, suggesting distinct variations in surface charges and electrostatic interactions. To contextualize these results, we compared the zeta potential of these nanoparticles with that of an HPAM solution, also recorded for three runs performed at URIL. HPAM is commonly used as a synthesis polymer in flooding experiments for oil recovery applications [28,29,30,31]. Zeta potential values for HPAM were determined as −36.2, −24.7, and −20.5 mV for the three successive measurements, resulting in an average zeta potential of −27.13 mV. This average potential value of HPAM is found to be close to the value obtained by Hu et al. [32]. In this study, it was remarked that the estimated potential value for HPAM dispersion is −32 ± 1.0 mV. This indicates that the stability of HPAM molecules is better than the CSNPs combination, but the stability of PYNPs is better than the two (both HPAM and CSNPs). Therefore, PYNPs nano-polymer mixed with HPAM gave magnificent results in oil recovery experiments conducted by Al-Jaber et al. [20]. The comparison reveals intriguing insights into the varying stability and surface charge among these two kinds of nanoparticles and HPAM. A tabular form for the results of the zeta potential for these solutions is demonstrated in Table 1.

**Figure 4:**
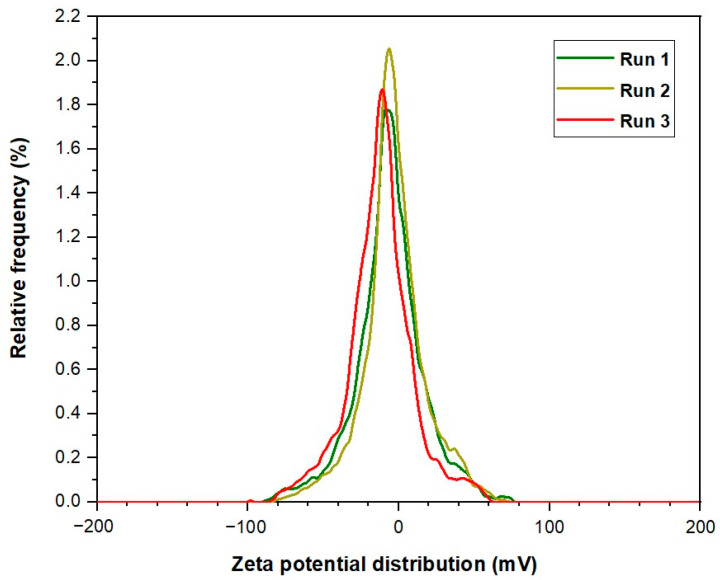
Zeta potential distribution for CSNPs.

**Figure 5:**
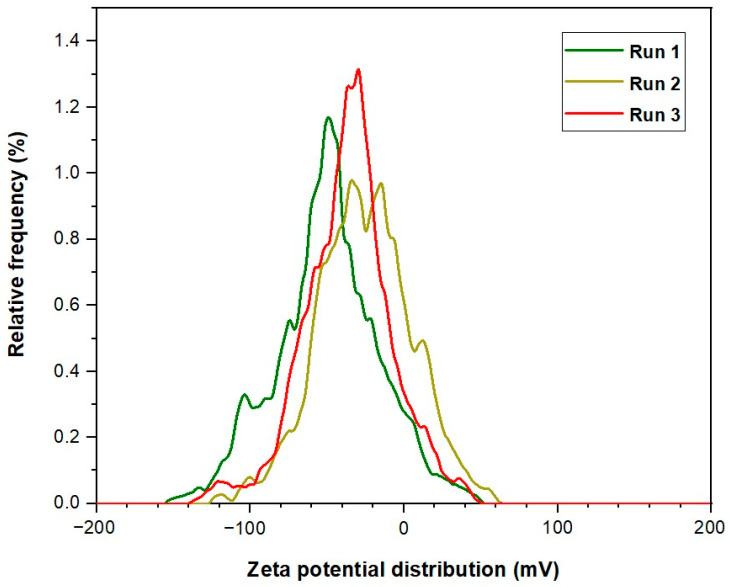
Zeta potential distribution for PYNPs.

**Figure 6:**
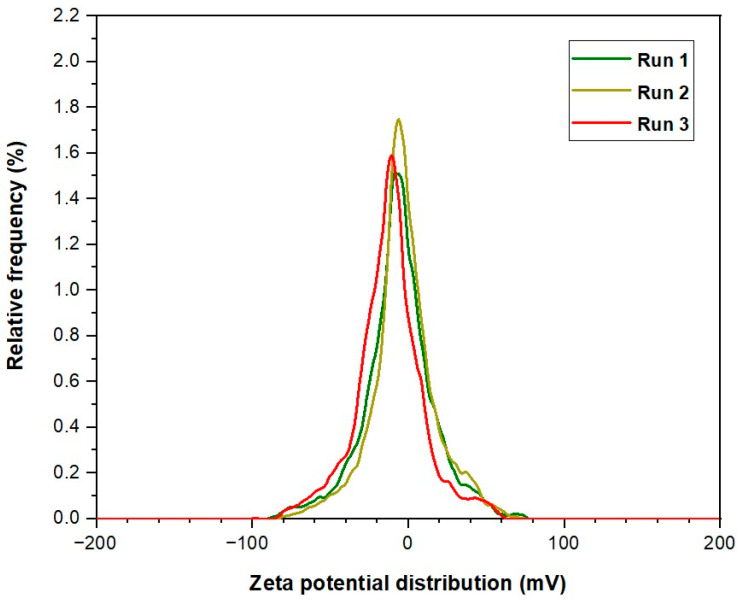
Zeta potential distribution for HPAM solution.

PYNPs and HPAM exhibit distinctive surface charge properties, influencing their dispersibility, aggregation behavior, and potential interactions with the surrounding media. These findings underscore the significance of zeta potential analysis in nanoparticle characterization, aiding in the optimization of nanomaterial applications across the oil industry. The observed variations in zeta potential highlight the importance of understanding surface charges in nanoparticle-based systems. The zeta potential curves constructed at these runs for CSNPs, PYNPs, and HPAM polymer are shown in Figure 4, Figure 5 and Figure 6, respectively.

### 2.3. Inspected Surfactants and Their Role in Wettability Alteration

The exploration into wettability alterations in Buff Berea core samples, after undergoing saturation with crude oil sourced from the Langgak oilfield, Sumatra, has unfurled the possible scenarios into the dynamics of reservoir behavior. The investigation, driven by the precision of contact angle measurements through the utilization of an optical instrument, revealed the following: the initial contact angles outstripped the 70° threshold towards 100°, which is close enough to the critical limit of 90 (fully oil-wet condition). This initial revelation stood as a testament to the successful saturation of core samples with crude oil (after it had dealt with Fsol to decrease its viscosity), thereby endorsing a good oil-wet nature for the inspected cores. In the subsequent phase, the core samples were subjected to soaking with a range of surfactant solutions at 60 °C, thoughtfully procured from PT SPR Langgak. As the core samples came into contact with the diverse array of surfactant treatments, the contact angles embarked on a remarkable descent, consistently below the critical 90° mark. What makes this alteration intriguing is the extent of this reduction, with some contact angles plummeting to levels as low as the twentieth-degree range, as listed in Table 2. The transition from an oil-wet orientation to water-wet conditions is not just noteworthy but paradigm-shifting, heralding enhanced potential for fluid flow and oil extraction [30].

The temperature choice, seamlessly aligned with the conditions prevalent in the Langgak oilfield in Sumatra, unlocks an understanding of wettability behavior within a framework that resembles actual field settings. The congruence between experimental circumstances and genuine conditions of the reservoir is a crux, ensuring that the findings gleaned are both meaningful and directly translatable to the pragmatic sphere of oil production. The shift from an oil-wet to water-wet propensity, validated by a pronounced reduction in contact angles, augments the efficacy of fluid flow and holds the promise of escalated recovery during oil extraction. As seen from Table 2, each core sample was dedicated to a certain surfactant solution for three days after all core samples had been soaked with oil for three days. The core specimen was sectioned into six small circular pieces, each exhibiting a diameter of 1.5 inches and a length of 0.5 inches.

### 2.4. Structural and Functional Analysis for NPs by XRD Analysis

XRD spectra obtained for both CSNPs and PYNPs exhibited distinct patterns that shed light on their crystallographic attributes. For CSNPs, the resulting XRD sketch showcases some sharp and well-defined diffraction peaks. These peaks correspond to the unique arrangement of atoms within the crystalline lattice of the nanoparticles. Conversely, the XRD sketch for PYNPs portrayed nearly the same pattern picture. The diffraction peaks in this pattern appeared less well-defined compared to CSNPs. This phenomenon is indicative of a lower degree of crystallinity, as illustrated later, and a more amorphous nature of the PYNPs in comparison to CSNPs.

**Figure 7:**
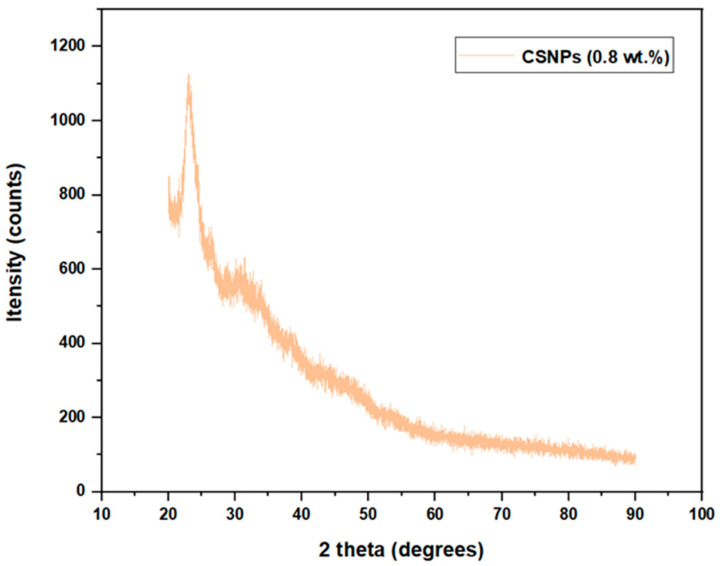
X-ray Diffraction pattern for CSNPs starch.

**Figure 8:**
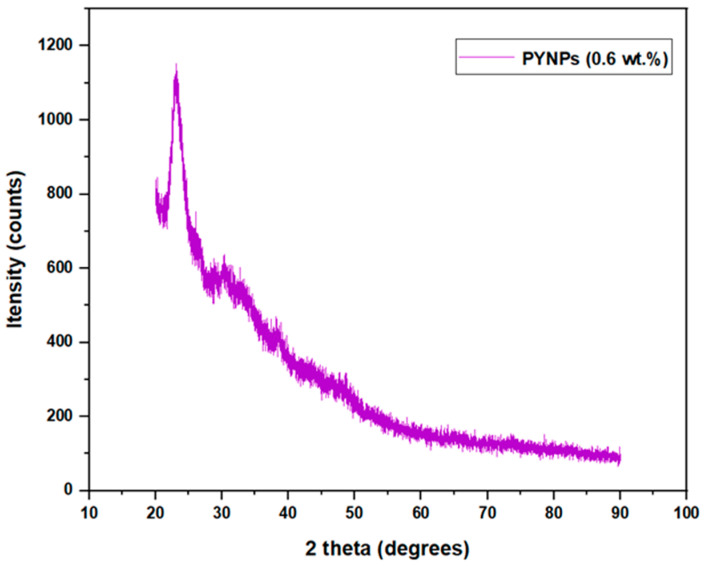
XRD pattern for PYNPs starch.

Both CSNPs and PYNPs exhibited a typical A-type crystalline pattern, which refers to a specific arrangement of atoms, molecules, or ions that display a distinct structure characterized by regular and repeating intermolecular distances. This pattern is commonly found in certain types of crystalline substances, especially in layered or lamellar structures. In an A-type crystalline pattern, the layers are typically stacked in a way that creates a relatively small interlayer spacing, resulting in a closer arrangement of atoms or molecules within each layer [33,34]. An A-type crystalline pattern was noticed at approximately 2θ = 20°, 23.1°, 26.4°, and 31.4° for CSNPs, as seen in Figure 7, whereas for PYNPs, an A-type was noticed at 2θ = 20.3°, 23°, and 30.4°, as seen in Figure 8.

The values of 2θ for CSNPs show that the crystallinity structure almost remained the same and did not change during the preparation period, but not for PYNP molecules. Similar trends for crystallinity were reported in some previous investigations [35,36,37]. As per preceding investigations, it was observed that the ultrasonication treatment had a significant impact on the disruption of the amorphous region of the starch compared to the crystalline region [38,39]. As explained later, the degree of relative crystallinity for CSNPs was 24.15%, which is higher than that of PYNPs (20.68%). The instability of the layered structure of starch present in PYNP molecules may be responsible for the reduction in relative crystallinity for this nanoparticle polymer.

### 2.5. Calculating the Relative Degree of Crystallinity for CSNPs and PYNPs

The degree of crystallinity is a crucial parameter that defines the proportion of crystalline regions within a material’s structure. In the case of CSNPs and PYNPs, X-ray diffraction (XRD) analysis was employed to ascertain their crystalline nature, as shown in the previous figures. The resulting XRD patterns exhibited characteristic peaks corresponding to specific crystal planes, enabling the determination of the relative degree of crystallinity for both CSNPs and PYNPs.

The acquired XRD data revealed that CSNPs exhibit a relative degree of crystallinity of approximately 24.15%, as shown below, while PYNPs display a relatively lower degree of crystallinity at around 20.68%. This disparity in crystallinity can be attributed to the inherent composition and molecular arrangement of the two starch sources [40,41]. Cassava starch, with its well-organized amylopectin and amylose structures, contributes to a higher degree of crystallinity. Conversely, purple yam starch, characterized by its irregular molecular arrangement, leads to a comparatively lower degree of crystallinity. Understanding the relative degree of crystallinity is pivotal as it influences the physical and chemical properties of the nanoparticles. The extent of crystallinity affects the nanoparticles’ thermal stability, mechanical strength, and interaction with surrounding environments [42,43].

Using the Origin application and the data supplied from the College of Science, UTM, that are related to the XRD analysis, the necessary steps that were followed to calculate the degree of crystallinity for both CSNPs and PYNPs were composed of drawing the relation between 2θ (x-coordinate) measured in degrees and Intensity (y-coordinate) measured in units of counts/sec through Origin, and these parameters were obtained from the XRD analysis. The next step was to calculate the area under the crystalline region for each starch kind, and this area was found between the crystalline peaks that were located on the resulting curve. To calculate this area, we tabbed Analysis on Origin software, then chose Peaks and Baseline, and we selected Peak Analyzer. After that, the next option was tabbing Open Dialogue, and a pop-up window appeared, from which the Baseline mode was changed to User Defined. After pressing Next and moving on to the Create Baseline category, we chose Add to add arbitrary Baseline Anchor Points.

The next stage was related to modifying these points by tabbing on the Modify/Del button and relocating these anchor points so that the new position was located at the down edge of the resulting curve. After that, the function of Enable Auto Find was disabled to manually find the peak values by pressing Add in the side window that appeared. After that, the area for the peak regions was calculated automatically by the software, and it was possible during this stage to manually adjust these areas by using the mouse cursor to include any unspecified region not located previously.

At the current point, the crystalline areas were calculated as demonstrated above, and the next step was to calculate the total area of the crystalline and amorphous regions. The same procedure of programming was repeated, and this time, there were only two differences that needed to be made. The first related to the choice of the baseline mode, which was set this time to Custom. We identified a horizontal line that was aligned with the lower edge of the curve. The second difference related to the selection of the peak values; in the present stage, only one peak was located in the curve, and the total area under the curve around this peak was selected to include the crystalline and amorphous regions. After obtaining the total area under the curve by the software, the relative degree of crystallinity for both CSNPs and PYNPs could be calculated by applying Equation (1). The degree of crystallinity calculated by this procedure is of high accuracy, and this method for calculating it is demonstrated by inserting the area of the crystalline region and that of the total area in an Excel program. As the field of nanotechnology continues to evolve, this method for calculating the crystallinity percent contributes valuable information to the ever-expanding knowledge of starch-based nanoparticles and their potential involvement in the oil industry as strong alternatives to synthesized polymers.

The impact of crystallinity on flood formation in nanoparticle-ASP oil recovery processes can be significant. Crystallinity refers to the degree to which a material exhibits a crystalline structure, which is characterized by ordered and repeating atomic or molecular arrangements. In the context of nanoparticles used in ASP flooding, crystallinity can affect several aspects of the process:

1. Nanoparticle performance: The crystalline structure of nanoparticles, such as those derived from starches like purple yam or cassava, can influence their performance as mobility control agents. Crystalline nanoparticles may have different interactions with the reservoir rock and fluids compared to amorphous nanoparticles;
2. Nanoparticle stability: Crystalline nanoparticles may exhibit different stability characteristics under reservoir conditions. Their ability to maintain their structural integrity in the presence of high temperatures, pressures, and chemical environments can impact their effectiveness in altering the reservoir’s fluid behavior;
3. Nanoparticle transport: Crystallinity can affect the transport of nanoparticles through porous reservoir rock. The size and shape of crystalline nanoparticles may influence their ability to penetrate and plug certain pore spaces, which can impact the efficiency of mobility control;
4. Chemical interactions: Crystalline nanoparticles may have distinct chemical interactions with ASP components, including surfactants and polymers. These interactions can affect the overall performance of the ASP flooding process.

In summary, crystallinity plays a role in the behavior and performance of nanoparticles in ASP flooding for enhanced oil recovery. Its impact can vary depending on the specific characteristics of the nanoparticles and the reservoir conditions. Understanding and controlling crystallinity are essential aspects of optimizing the effectiveness of nanoparticle-ASP flooding processes.

### 2.6. Modified ASP Flooding versus Conventional ASP Flooding

ASP viscosity, as a standalone parameter, may not provide comprehensive insights into enhancing overall oil recovery. Measuring ASP viscosity would not necessarily yield data directly linked to increased oil recovery.

Polymers in ASP formulations primarily serve as mobility control agents. Their central role lies in enhancing the viscosity of the injected fluid, thereby improving reservoir sweep efficiency and conformance control. While ASP viscosity as a whole is important, the contribution of the polymer component to overall viscosity is often more substantial. Therefore, focusing on polymer concentration and viscosity offers a targeted approach to optimizing ASP flooding performance.

In this study, the ASP flooding process was investigated without the incorporation of nano-starch, specifically CSNPs and PYNPs. The surfactants used were provided by the PT SPR Langgak Company in Sumatra. The outcomes of the ASP flooding experiments highlighted significant distinctions between the ASP flooding with and without the inclusion of these starch nanoparticles. To better understand these findings, a comparative analysis was conducted with the results reported by Al-Jaber, H.A. et al. [20], as they explored the influence of CSNPs and PYNPs on ASP flooding.

The current study compared three combinations that yielded the highest oil recovery in that work with a similar formulation, but the polymer consisted of only HPAM at a concentration of 2000 ppm. By conducting flooding experiments exclusively with HPAM alone, the investigation aimed to assess whether oil recovery can still be enhanced in the absence of starch nanoparticles. Stability analysis demonstrated that CSNPs and PYNPs remained dispersed within the solution, but PYNPs were more stable and active, as shown from zeta distribution tests. This stability is crucial in ensuring consistent nanoparticle distribution throughout the solution, thereby averting potential clogging of pore networks. The study raised an essential question: Could close results be achieved using only HPAM without nanoparticles? Addressing this question was essential to understand the specific contribution of nano-starch to the ASP flooding process.

It was found by Al-Jaber, H.A. et al. [20] that the most effective combinations that resulted in higher oil recovery were specifically the combinations (NaOH—PSC EOR 2.2—(HPAM + PYNPs)), with an incremental oil recovery of 39.17%; (NaOH—PSC HOMF—(HPAM + CSNPs)), with an incremental oil recovery of approximately 35%; and also (NaOH—PSC HOMF—(HPAM + PYNPs)), which resulted of third higher recovery of about 34.61%. Therefore, these three combinations were selected for the conventional ASP flooding in this study, with the polymer component consisting solely of HPAM.

Upon initiating conventional ASP flooding utilizing the first formulation NaOH (1.28 wt.%), PSC EOR 2.2 (0.98 wt.%), and HPAM (0.2 wt.%), a noteworthy incremental oil recovery of approximately 20.8% OOIP was achieved. This outcome demonstrated a decrease of roughly 18.37% compared to ASP flooding encompassing HPAM with PYNPs (0.60 wt.%). The combination that consisted of NaOH (1.28 wt.%), PSC HOMF (0.63 wt.%), and HPAM (0.2 wt.%) yielded an incremental oil recovery of 24.24% OOIP. This achievement displayed a reduction of around 10.76% when compared to the analogous combination that involved HPAM accompanied by CSNPs (0.8 wt.%). Also, the last combination revealed a reduction of about 10.37% when compared to the same ASP formula that contained HPAM integrated with PYNPs (0.6 wt.%).

**Figure 9:**
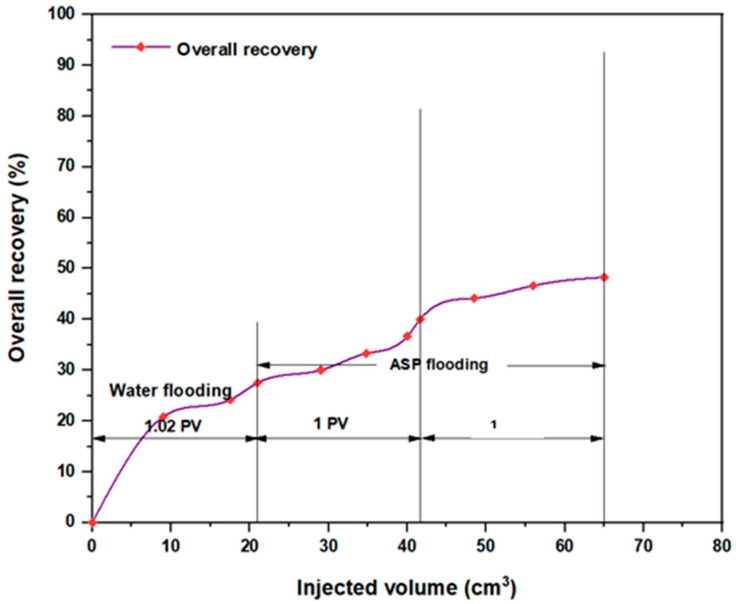
Overall oil recovery from water and ASP flooding. ASP combination consisted of NaOH (1.28 wt.%), PSC EOR 2.2 (0.98 wt.%), and HPAM (0.2 wt.%) at 60 °C.

**Figure 10:**
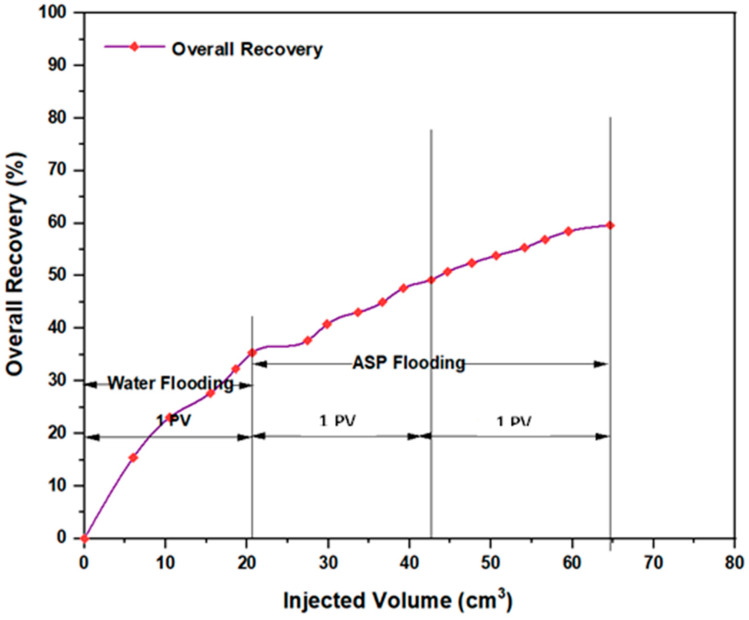
Overall oil recovery from water and ASP flooding. ASP combination consisted of NaOH (1.28 wt.%), PSC HOMF (0.63 wt.%), and HPAM (0.2 wt.%) at 60 °C.

However, it remains evident that performing ASP flooding with its current components and the same concentrations as those performed in the compared study has helped in obtaining acceptable recovery fractions, as shown by numbers. Anyway, by referring to that study, additional empirical validation has been provided through our study. Figure 9 and Figure 10 demonstrate the overall oil recovery achieved through the implementation of conventional ASP, and their comparisons to the aforementioned study are clarified in Table 3 below.

As seen from this study, ASP flooding without starch nanoparticles involved the traditional application of alkalis, surfactants, and polymers. When examining the recovery results, it is evident, as shown in Table 2, that ASP flooding with the inclusion of PYNPs exhibited a higher recovery rate compared to CSNPs. Anyway, the recovery rates for both CSNPs and PYNPs are impressive, surpassing the baseline of ASP flooding adopted in this study. The enhanced recovery achieved through ASP flooding with PYNPs can be attributed to the distinct properties of these nanoparticles. PYNPs possess a large surface area, allowing for better interaction with oil molecules and reservoir surfaces [20]. This enhanced interaction facilitates the displacement of trapped oil, leading to a higher recovery rate. Additionally, the PYNPs’ particles aid in decreasing the IFT between oil and water, enabling better oil mobilization [44,45]. It is important to note that while CSNPs also contributed to increased oil recovery, their smaller size compared to PYNPs may limit their efficiency in certain conditions [46]. The key lies in the balance between nanoparticle size, achieved wettability alteration, and interfacial tension reduction [47,48].

Based on the advantages of crystallinity, as shown in Table 4 [26], as shown below, it becomes imperative to consider CSNPs within the ASP formulation rather than PYNPs due to their higher degree of crystallinity. This is because higher crystallinity presents a multitude of advantages that cannot be overlooked. The degree of crystallinity for CSNPs in this study was calculated at 24.15%, whereas it was only 20.68% for PYNPs. Consequently, PYNPs might not be suitable for reservoirs subjected to challenging conditions such as elevated temperature and high salinity degrees.

When exposed to higher temperatures, amorphous materials might undergo a softening transition and transformation into distinct forms. Furthermore, PYNPs, as amorphous material, exhibited a short-range structural arrangement because of their irregular nature. Despite valuable insights given by the zeta potential, which indicates the stability of PYNPs (measured at −36.33 mV) in comparison to CSNPs (measured at −10.68 mV), a fraction of uncertainty is available when considering their integration into the polymer component. Moreover, the difference in incremental oil recovery achieved by adding these starch nanoparticles in comparison to CSNPSs amounts to a mere 4.17% from the highest recovery of PYNPs and CSNPs. From a pragmatic standpoint, this margin is not very significant. CSNPs, at a concentration of 0.80 wt.%, emerge as the optimal starch component for a secure amalgamation with HPAM, particularly given the insights into crystallinity and their preference in any process. This amalgamation holds the potential to yield the most promising recovery and adds important insights into the exploitation of matured reservoirs.

## 3. Materials and Methods

### 3.1. Materials

#### 3.1.1. Buff Berea Core Samples

**Figure 11:**
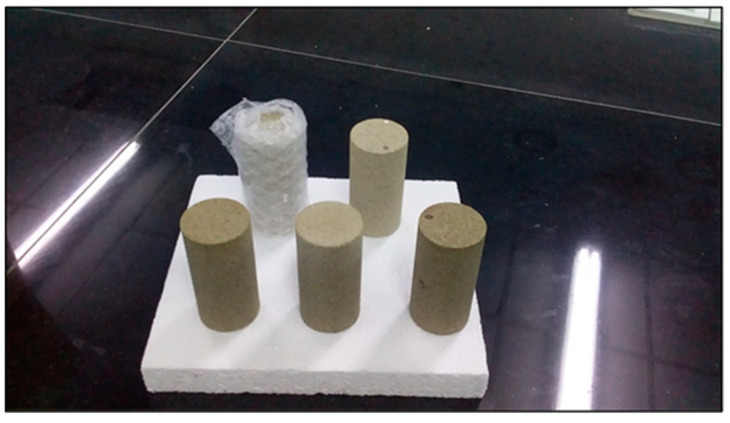
Buff Berea core samples used in flooding experiments.

Five Buff Berea core samples were received from Atama Tech Sdn. Bhd. These samples, which had the shape of circular rods, were utilized in test experiments related to water and ASP flooding. The core samples (shown in Figure 11) had similar characteristics to those of sandstone layers located in the Langgak oilfield in Sumatra. Each core sample was 3 inches in length and had a diameter of 1.5 inches.

#### 3.1.2. Heavy Oil

A shipment of heavy oil with 31.9° API from the Langgak oilfield in Sumatra, Indonesia, was supplied for the current study. This crude oil exhibited a viscosity of approximately 43.668 cp, and it solidified at normal temperatures (25 °C). The pour point and wax content of most Indonesian crude oils generally lie within 35 to 40 °C and 20% to 25%, respectively. To address such crude oil, specialized technologies are essential to maintain this oil in a liquid state. To achieve this, the oil underwent treatment by employing a chemical solution known as Fsol at a ratio of 1:1. The procurement of the chemical solution used was made through Innochems Technologies Sdn. Bhd. in Johor, Malaysia.

The chemical solution Fsol possesses the ability to reduce the viscosity of this heavy oil and transform it into a liquid state at ambient temperature while preserving its essential characteristics. To achieve this, the crude oil was mixed with the aforementioned solution in the specified ratio and then subjected to stirring with a magnetic stirrer for approximately 10 min. To guarantee a uniform resulting mixture for treated oil, it was further subjected to heating at a temperature of 60 °C in an oven for approximately 30 min prior to utilization in core samples for water and ASP flooding.

#### 3.1.3. Synthesized HPAM

HPAM, the polymer most employed in EOR processes, has gained widespread recognition due to its consistent viscosity within the low- to moderate-temperature range (approximately 693 to 787 mPa·s at 35 °C), coupled with its chemical and physical stability. HPAM utilized in this research had a molecular weight of 27.7 × 10^6^ g/mol, deemed appropriate for a variety of EOR applications, procured from Tricell Bioscience Resources Co., Johor, Malaysia. A 0.2% (*w*/*w*) aqueous solution (brand R&M) was included in ASP flooding without adding natural biopolymers like cassava or purple yam starches.

#### 3.1.4. Sodium Hydroxide (NaOH) and Sodium Carbonate (Na_2_CO_3_)

In this particular research, two distinct alkalis, NaOH (1.28 wt.%) and Na_2_CO_3_ (0.90 wt.%), were examined. The provider of NaOH was ASIA (QREC) Sdn. Bhd. located in Selangor, Malaysia, while Na_2_CO_3_ was procured from ASIA CHEMIE (QREC) Co., Ltd. in Huai Kapi, Thailand.

#### 3.1.5. Surfactants Brought from the PT SPR Langgak Company

The PT SPR Langgak Company in Sumatra, Riau, provided four kinds of surfactants with specific concentrations. However, as observed from the study performed by Al-Jaber, H.A. et al. [20], not all supplied surfactants performed well in obtaining higher oil recovery. The company’s surfactants investigated in the current study included PSC HOMF (0.63 wt.%), PSC EOR 2.2 (0.98 wt.%), Mits-5L001 (1.0 wt.%), and Dekasurf SF 9136 (1.24 wt.%).

#### 3.1.6. Native Cassava Starch

Native cassava starch weighing 1 kg was procured from a local market. Cassava, a highly versatile vegetable with global consumption, is a primary source of tapioca starch. After undergoing dehydration and drying processes, tapioca was transformed into a white powder referred to as cassava starch. Cassava starch NPs (CSNPs) utilized in the flooding experiments possessed an average particle size of 52.92 nm, and their concentration in these experiments was 0.8 wt.% (*w*/*w*) [21].

#### 3.1.7. Purple Yam Starch

Purple yam tubers weighing eighteen kilograms were purchased from a local market in Johor. Scientifically known as ‘*Dioscorea Alata*’, and also grater yam or water yam, this particular yam species, along with others, has been cultivated and farmed for its starchy tubers across Southeast Asia and New Guinea. The flooding experiments utilized purple yam NPs (PYNPs) made from these tubers with an average particle size of 363.12 nm and a concentration of 0.6 wt.% (*w*/*w*) [21].

### 3.2. Methods

#### 3.2.1. Characterization Techniques for Produced Starch Nanoparticles

The yield of produced nanoparticles was 90.53% for CSNPs and 85% for PYNPs [21]. Produced starch nanoparticles were evaluated by performing transmission electron microscopy (TEM), Fourier-transform infrared spectroscopy (FTIR), and zeta potential distribution. Results from TEM analysis showed that the particles were well-distributed, and an abundance of small nano-size for the two species was generated during the preparation process. TEM analysis for these NPs showed a diverse appearance of these particles distributed between spherical, hexagonal, and rod-like shapes. This intriguing formation highlights the versatility of generation for these particles.

**Figure 12:**
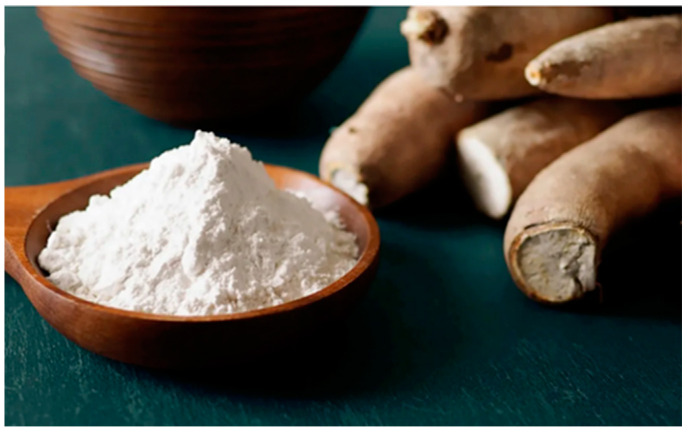
Sample of CSNPs starch.

**Figure 13:**
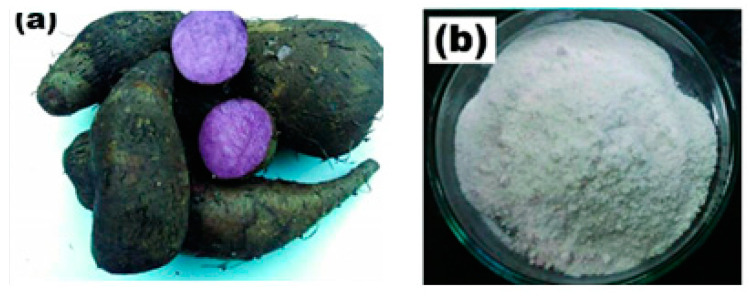
Sample of PYNPs starch. (**a**) purple yam roots, (**b**) extracted starch.

The method applied for calculating the particle sizes of these NPs depends on intensity percentage, which is particularly valuable in identifying any minor degree of particle aggregation. For zeta potential analysis for these starch nano-polymers, three runs were performed for each kind to calculate the zeta potential. Its value gives an indication of the charge stability on the surface of these particles, and when its value is increased, the particles are more stable in the solution and will not aggregate. Table 5 shows the stability value ranges and their relation to the stability criterium [49]. The mean zeta potential for CSNPs was −10.7 mV. This means these particles were critically stable in the polymer formation according to the stability mechanism. The value of zeta potential for PYNPs was at the −36.3 mV level. Thus, the stability for PYNPs was higher than CSNPs, and this means that the particles for this component will not aggregate [50,51]. Figure 12 and Figure 13 show fresh samples of these nano-polymers.

#### 3.2.2. Size Analysis for Produced Nanoparticles

The final weight of the produced nanoparticles was found to be 36.21 g for CSNPs and 34 g for PYNPs. Based on that, the yield of the produced nanoparticles from cassava starch (CAS) and purple yam starch (PYS) was 90.53% and 85%, respectively [21].

Particle size assessment through TEM analysis provided essential data about the size and shapes (spherical, hexagonal, or longitudinal) of the produced particles. The evaluation was carried out at the University Industry Research Laboratory (URIL). To determine the optimal concentration for PYNPs and CSNPs, various concentrations (0.2, 0.4, 0.6, 0.8, and 1 wt.%) were tested. Each nanoparticle material was prepared at the above concentration by dissolving it in a brine solution with the same salinity as the Langgak oilfield’s formation water (100 ppm). Subsequently, 2000 ppm of HPAM was added to each nanoparticle solution to form a “hybrid or nano-polymer”. The hybrid polymers were then exposed to paraffin oil at 60 °C to replicate the crude oil from the Langgak oilfield. The IFT between the paraffin oil and the hybrid polymers was measured using a KRUSS EasyDyne tensiometer (K20). The IFT of the hybrid polymers with paraffin oil was evaluated to determine the optimum concentration of these biopolymers for use in flooding experiments. The IFT was evaluated in relation to the nanoparticle (NP) concentration in the hybrid polymer [21]. The assessment indicates that as the NP concentration increases, the IFT decreases until it reaches a “minimum value”. The concentration of NPs at this minimum value represents the optimal concentration for that nanoparticle solution in the combined polymer.

#### 3.2.3. Surface Charge Measurement for Produced Nanoparticles

Zeta potential distribution is a crucial parameter used to assess the stability of any colloidal particles in a solution. It provides valuable information about the surface charge of particles and their interactions with other particles in the system. Zeta potential is a measure of the potential difference between the surface of the particles and the surrounding liquid phase.

The charge stability of CSNPs and PYNPs in the polymer solution can be assessed by analyzing the zeta potential distribution for these particles. By using the electrophoretic light scattering technique, the velocity of the particles is measured by tracking the frequency shift of the light scattered during their motion. If the absolute zeta potential exceeds 30 mV, this indicates strong repulsive forces between these particles, leading to stability and well-dispersed behavior for particles [52]. Conversely, if the values are lower than 10 mV then weaker repulsive forces occur, and as a result, these particles become more prone to agglomeration and instability [52].

#### 3.2.4. Soaking of Buff Berea Core Samples

To assess the wetting properties of tested surfactants, Buff Berea core samples were employed for this regard. The Langgak oilfield’s crude oil was mixed with Fsol in a 1:1 ratio to lower its viscosity and maintain its liquid state at normal temperature. To ensure uniform composition for this treated oil, it was stirred by a magnetic stirrer for 10 min and then placed in an oven at 60 °C for around 30 min before using it further. The core samples were placed in contact with this liquified crude oil for around three days. This step was necessary to ensure complete saturation for those cores with crude oil. At this stage, the wettability of core samples was measured through an optical contact angle instrument. The type of instrument was an OCA 15EC produced by Dataphysics, which is a part of Anton Paar Group, a global company with its main headquarters located in Filderstadt, Germany. This instrument has the ability to measure the contact angle and drop shape analysis for different samples. This model also offers great convenience as it can be easily disassembled and transported in the optional carrying case.

**Figure 14:**
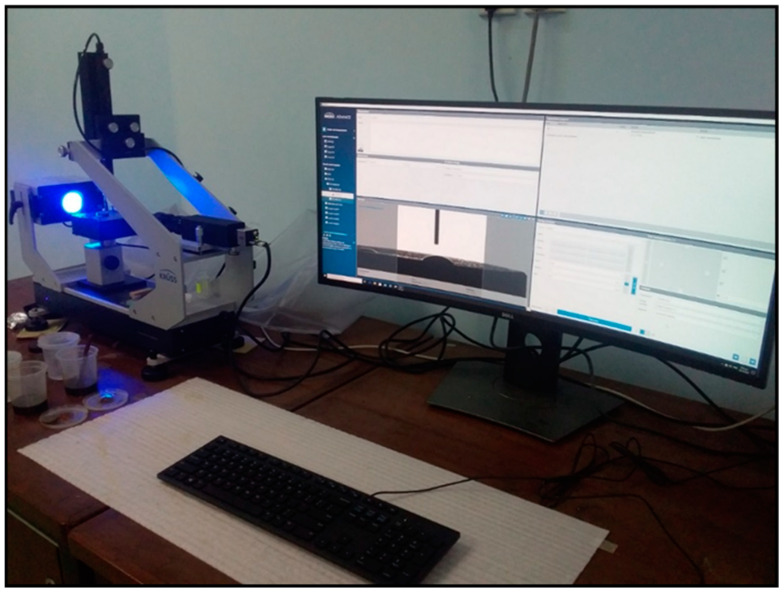
Optical instrument (OCA 15EC) used in measuring the contact angle for soaked sandstone cores with oil and surfactants.

The measured contact angle values should be higher than 90°, which indicates that the core samples were fully saturated with crude oil (oil-wet). After that, each kind of surfactant at its optimal concentration was placed in contact with these cores in such a way that those cores were totally immersed in these solutions. The Vessels that contained these solutions with core samples were placed in an oven at 60 °C to for three days for stability and saturation. To examine the new wettability for the core samples that were soaked with these surfactants, the contact angle was computed again using the optical instrument, as shown in Figure 14. The new contact angle values must be less than 90° (water-wet) for successful application of these solutions. When these conditions are met, this is considered beneficial for ASP combination with its current components (surfactants, nano-polymers, and alkalis).

#### 3.2.5. Finding the Crystallinity Degree for CSNPs and PYNPs Nano-Starches

The degree of crystallinity for any substance can be divided generally into two types: crystalline and amorphous substance. Crystalline solids exhibit distinct boundaries and surfaces and display high melting points. On the other hand, amorphous solids possess irregular or curved surfaces and undergo progressive melting across a broad temperature range [53].

As per the advantages of crystallinity, it was crucial to attain crystalline forms for cassava and purple yam particles to ensure their optimal performance in flooding experiments. Additionally, the nanoparticles’ melting point must be sufficiently high and in a well-defined range to suit the Langgak oilfield’s temperature of around 60 °C. To precisely determine the crystallinity degree for the produced nanoparticles, the Origin interface can be utilized to calculate the area under the curve for the crystalline regions, which are characterized by sharp peaks. Also, the amorphous area can be found by a similar procedure, and the total area under the curve can be estimated through the application. The primary data that need to be known for calculating the area under these two regions can be obtained from X-ray diffraction (XRD) analysis through a D8 ADVANCE X-ray diffractometer (Bruker, Karlsruhe, Germany). XRD is a technique that offers detailed insights into the materials’ crystallographic structure, chemical composition, and physical properties. The XRD analysis was performed at the labs of the College of Science, UTM University.

The crystalline percent can be estimated by integrating the areas under the crystalline region (Ac) and the amorphous region (Aa) that can be calculated from Origin through the obtained data from XRD analysis according to the following equation [54]:

Xc = (Ac/(Ac + Aa)) × 100%(1)

where Xc is crystallinity (percent), Ac is the crystalline area (degrees), and Aa is the amorphous area (degrees).

#### 3.2.6. Flooding Tests

Buff Berea sandstone core samples were vacuumed and positioned in a saturation vessel. The vacuum process lasted approximately three hours to eliminate air from the core samples. A brine solution with 100 ppm salinity was created by dissolving 10 g of NaCl in 1000 mL distilled water and was introduced into the saturation vessel via the vacuum pump. As the brine filled the vessel, the excess quantity was discharged into a conical flask. Gradually, the pressure inside the saturation vessel was increased by injecting brine water at a rate of 8 cm^3^/min by using a Teledyne pump. Once the pressure reached around 2200 psi, brine injection ceased, and the saturation vessel maintained this pressure for 2–3 days to guarantee complete core saturation with brine.

Nitrogen gas was introduced into a confinement vessel to facilitate oil saturation and mimic original oilfield conditions. Once saturated with brine, the core samples underwent crude oil saturation, achieved by injecting the original crude oil (mixed with Fsol). The oil injection proceeded at a flow rate of 7 cm^3^/min until excess oil started to accumulate in a collection container. The volume of water collected in the container was equal to the volume of OOIP injected into the core. Following core saturation with oil, water flooding (using 100 ppm brine) commenced at a flow rate of 5 cm^3^/min. At intervals of roughly three minutes, the quantity of discharged water and oil (in terms of volume) resulting from water flooding was measured. The flooding persisted until one pore volume (PV) of water was introduced.

ASP flooding commenced immediately after the water flooding phase. The flow rate was set at 3–3.5 cm^3^/min, and the outputs were monitored every three minutes like before. The ASP formulation consisted of NaOH (1.28 wt.%) as alkalis and PSC EOR 2.2 (0.98 wt.%) or PSC HOMF (0.63 wt.%) as surfactant. Additionally, the polymer used was comprised mainly of HPAM (0.2 wt.%) without starch nanoparticles. To ensure a constant temperature of around 60 °C for both water and ASP flooding, the core sample, which was located inside the confinement vessel, was placed inside an electric oven at 60 °C. ASP flooding continued until 2 PV of cthe hemical solution was injected. The oil recovery fraction (%) (RF) for ASP flooding was computed using the below subsequent formula [55]:

RF = (Total volume of oil collected/OOIP) × 100(2)

## 4. Conclusions

This study included two scopes: The first related to the characterization of nanoparticles produced from two starches, CAS and PYS, through different analyses like TEM, FTIR, zeta potential, and XRD. The second scope related to performing ASP flooding using different alkalis, surfactants, and HPAM and comparing the outputs of this flooding with a study that utilized these nano-starches combined with HPAM. The purpose was to find the optimal ASP formulation that satisfied both oil recovery requirements and stability against harsh reservoir conditions like high temperature. This study was made to simulate the conditions of the Langgak oilfield in Sumatra but can be adopted for any reservoir that has similar conditions. Concerning zeta potential, PYNPs exhibited remarkable stability at −36.33 mV, outperforming both CSNPs (−10.68 mV) and HPAM (−27.13 mV).

Wettability changes in Buff Berea cores that were saturated with PT SPR’s surfactants at 60 °C after being saturated with crude oil revealed the transition from oil-wet to water-wet conditions. Substantial contact angle reductions indicate a shift toward water-wetness, boosting the oil extraction process. XRD analysis uncovered distinct crystallographic attributes of CSNPs and PYNPs, elucidating their structural properties. CSNPs exhibited well-defined diffraction peaks, portraying a higher degree of crystallinity compared to PYNPs. Both displayed a typical A-type crystalline pattern with layers stacked closely. CSNPs maintained their crystallinity, while PYNPs showed reduced crystallinity due to possible structural instability.

XRD analysis for CSNPs and PYNPs indicated distinctive patterns which represented crystalloid planes. CSNPs exhibited around 24.15% crystallinity, while PYNPs displayed approximately 20.68%. Crystallinity impacts important properties like thermal stability and interaction with surrounding materials. Utilizing Origin software, crystallinity was calculated using the area under the peaks and total curve area, providing accurate results for starch-based nanoparticles.

The concentration of 0.98 wt.% for PSC EOR 2.2 surfactant may indeed seem relatively high. However, the principle guiding this decision is rooted in the concept of Critical Micelle Concentration (CMC), as expounded upon in Reference [21]. CMC represents the point at which surfactant molecules in a solution begin to self-assemble into micelles, which is a key mechanism for enhancing oil recovery. This concentration is critical to achieving the desired performance in ASP (or surfactant) flooding.

In practical field applications, the choice of surfactant concentration involves a delicate balance between its effectiveness in reducing interfacial tension and the associated cost. While a higher surfactant concentration can yield superior results in terms of oil recovery, it can also lead to increased operational expenses. The decision to use a 0.98 wt.% concentration is left for the company that runs the oilfield, according to their financial sources and calculations, or modify it to lower acceptable margins (<0.98 wt.%).

In conclusion, ASP flooding experiments performed in its traditional fashion in this study showcased significant oil recovery variation when compared to another study employing CSNPs and PYNPs combined with HPAM. Despite the fact that PYNPs yielded higher recovery and considering CSNPs’ greater crystallinity (24.15% vs. 20.68% for PYNPs), making them more favorable for optimal ASP formulation, PYNPs’ amorphous nature might limit their suitability under harsh reservoir conditions. It is true that zeta potential favored PYNPs, but their impact on oil recovery was only 4.17% when compared to CSNPs. Thus, CSNPs (0.80 wt.%) confidently emerge as the best choice for biopolymer, ensuring a promising recovery in oil production when they merge with HPAM.

## Figures and Tables

**Table 1:** Results of zeta potential values for the three solutions and the average value.

Type of Solution	Zeta Potential (mV)
CSNPs	−9.3, −10.3, −12.4
Average for CSNPs	−10.68
PYNPs	−43.9, −33.8, −31.3
Average for PYNPs	−36.33
HPAM Solution	−36.2, −24.7, −20.5
Average for HPAM	−27.13

**Table 2:** Contact angle for six Buff Berea core samples soaked with crude oil and different sets of surfactants at 60 °C.

Core Samples	Immersing with Modified Crude Oil	Immersing with PSC HOMF (0.63 wt.%)	Immersing with Dekasurf SF 9136 (1.24 wt.%)	Immersing with Mits-5L001 (1.0 wt.%)	Immersing with PSC EOR 2.2 (0.98 wt.%)
	Contact Angle (in Degrees)				
Core 1	71.2	37.15	-	-	-
Core 2	75.2	-	36.8	-	-
Core 3	79.8	-	-	25.95	-
Core 4	82.7	-	-	-	26.15

**Table 3:** Differences in oil recovery between AL-Jaber, H.A. et al. [20] and the current study.

S.	Alkaline and Its Concentration (wt.%)	Surfactant and Its Concentration (wt.%)	Polymer and Its Concentration (wt.%)	Incremental Oil Recovery Obtained by Al-Jaber, H.A. et al. [20] (%)	Incremental Oil Recovery Obtained by This Study (%)	Difference in Oil Recovery between the Two Studies (%)
1	NaOH (1.28)	PSC EOR 2.2 (0.98)	(Mixed) HPAM (0.2) + PYNPs (0.6)	39.17	-	-
2	NaOH (1.28)	PSC EOR 2.2 (0.98)	HPAM (0.2) only	-	20.8	18.37
						
3	NaOH (1.28)	PSC HOMF (0.63)	(Mixed) HPAM (0.2) + CSNPs (0.8)	35.0	-	-
4	NaOH (1.28)	PSC HOMF (0.63)	HPAM (0.2) only	-	24.24	10.76
						
5	NaOH (1.28)	PSC HOMF (0.63)	(Mixed) HPAM (0.2) + PYNPs (0.6)	34.61	-	-
6	NaOH (1.28)	PSC HOMF (0.63)	HPAM (0.2) only	-	24.24	10.37

**Table 4:** Major differences between crystalline solids and amorphous solids.

Crystalline Solids	Amorphous Solids
Atoms are arranged in a regular, three-dimensional pattern.	The arrangement of atoms is irregular and lacks a specific pattern.
Crystalline substances exhibit a well-defined melting point, indicating the temperature at which they start to melt.	When subjected to heat, amorphous solids undergo a gradual softening process over a wide range of temperatures and can transform into various forms.
They exhibit a well-defined heat of fusion.	Their heat of fusion is not constant.
Crystalline solids are often referred to as true solids.	Amorphous solids are known as pseudo-solids or super-cooled liquids.
They exhibit long-range patterns for the arrangement of particles.	They exhibit short-range patterns for the arrangement of particles.

**Table 5:** Stability behavior of colloids according to the value of zeta potential.

Magnitude of Zeta Potential (mV)	Stability Behavior
0 to 5	Rapid coagulation of flocculation
10 to 30	Incipient instability
30 to 40	Moderate stability
>61	Excellent stability

## Data Availability

Not applicable.

## Nomenclature

ASP	Alkaline–surfactant–polymer
CAS	Cassava starch
PYS	Purple yam starch
CMC	Critical micelle concentration (wt.%)
Ac	Crystalline area, degrees
Aa	Amorphous area, degrees
Xc	Relative crystallinity, %
cc	Cubic centimeter
cp	Centipoise
CSNPs	Cassava starch nanoparticles
Dekasurf SF 9136	Special surfactant
EOR	Enhanced oil recovery
FTIR	Fourier transform infrared spectroscopy
Fsol	F solution
HPAM	Partially hydrolyzed polyacrylamide
IFT	Interfacial tension, mN/m
Mits-5L001	Special surfactant
NaO	Sodium hydroxide
Na_2_CO	Sodium carbonate
NPs	Nanoparticles
NP	Nanoparticle
OOIP	Original oil in place
PV	Pore volume, cm^3^
pp	Part per million
PSC HOMF	Special surfactant
PSC EOR 2.2	Special surfactant
PYNPs	Purple yam nanoparticles
Q	Volumetric flow rate, cm^3^/min
RF	Oil recovery fraction, %
SFT	Surface tension, mN/m
SiO_2_	Silicon dioxide
TiO_2_	Titanium dioxide
TEM	Transmission electron microscopy
URIL	University Industry Research Laboratory
XRD	X-ray diffraction

## References

[1] Wang X., Wang F., Taleb M.A.M., Wen Z., Chen X. (2022). A Review of the Seepage Mechanisms of Heavy Oil Emulsions during Chemical Flooding. Energies.

[2] Editors G., Shiun Lim J., Shin Ho W., Klemeš J.J., Husein N., Yunan M.H., Ismail I., Rosli Wan Sulaiman W., Boyou N.V. (2018). Enhanced Oil Recovery by Alkaline-Surfactant-Polymer Alternating with Waterflooding. Chem. Eng. Trans..

[3] Hongyan C., Jie C., Jian F., Hexin L., Qing W., Wenli L. ASP flooding: A solution for chemical enhanced oil recovery in high temperature, low salinity reservoir. Proceedings of the Society of Petroleum Engineers—SPE Kingdom of Saudi Arabia Annual Technical Symposium and Exhibition 2018.

[4] Huang M. (2020). Recovery Characteristics of Weak Alkali ASP Flooding in Second Oil Reservoir. IOP Conf. Ser. Earth Environ. Sci..

[5] Li Y., Kong B., Zhang W., Bao X., Jin J., Wu X., Liu Y., Wang Y., He X., Zhang H. ASP flood with novel mixtures of anionic-cationic surfactants for high water cut mature sandstone reservoir: From laboratory to field application. Proceedings of the SPE Middle East Oil and Gas Show and Conference, MEOS, Proceedings.

[6] Khan M.Y., Samanta A., Ojha K., Mandal A. (2009). Design of alkaline/surfactant/polymer (ASP) slug and its use in enhanced oil recovery. Pet. Sci. Technol..

[7] Sheng J.J. (2014). A comprehensive review of alkaline–surfactant–polymer (ASP) flooding. Asia-Pac. J. Chem. Eng..

[8] Bera A., Kumar T., Ojha K., Mandal A. (2014). Screening of microemulsion properties for application in enhanced oil recovery. Fuel.

[9] Zuo X., Li S., Li W., Song R., Wang T., Xu H. A study on the remaining oil after strong base ASP flooding. Proceedings of the SPE Annual Technical Conference and Exhibition.

[10] Arvis A., Le Van S., Chon B.H. (2017). Feasibility study of alkali–surfactant–polymer flooding on enhancing heavy-oil recovery in a heterogeneous thin reservoir. Int. J. Appl. Eng. Res..

[11] Yin D., Zhao D., Gao J., Gai J. (2017). Experimental study of enhancing oil recovery with weak base alkaline/surfactant/polymer. Int. J. Polym. Sci..

[12] Guo H., Li Y., Wang F., Gu Y. (2018). Comparison of strong-alkali and weak-alkali ASP-flooding field tests in Daqing oil field. SPE Prod. Oper..

[13] Pei H., Zhang G., Ge J., Jin L., Ma C. (2013). Potential of alkaline flooding to enhance heavy oil recovery through water-in-oil emulsification. Fuel.

[14] Arsad A., Al-Jaber H.A., Junin R., Bandyopadhyay S., Abdulmunem A.R., Oseh J.O., Augustine A., Abdurrahman M.D., Kadir E.A., Rahim S.K.A. (2022). Recent advances in ASP flooding and the implementation of nanoparticles to enhance oil recovery: A short review. Pet. Sci. Technol..

[15] Yang P., Li Z.A., Xia B., Yuan Y.J., Huang Q.T., Liu W.L., Cheng C.Y. (2019). Comprehensive review of alkaline–surfactant–polymer (ASP) enhanced oil recovery (EOR). Springer Series in Geomechanics and Geoengineering Book Series (SSGG).

[16] Eseimokumoh I.B., Woyintonye I., Eniye O., Preye T.-A.N., Young E.E. (2021). Improving Oil Recovery Efficiency Using Corn starch as a Local Polymer for Enhanced Oil Recovery Processes. Int. J. Curr. Sci. Res. Rev..

[17] Cheng J., Zhou W., Wang Q., Cao G., Bai W., Zhao C., Luo M. Technical breakthrough in production engineering ensures economic development of ASP flooding in daqing oilfield. Proceedings of the Society of Petroleum Engineers—SPE Asia Pacific Oil and Gas Conference and Exhibition, APOGCE 2014, Changing the Game: Opportunities, Challenges and Solutions.

[18] Keykhosravi A., Vanani M.B., Aghayari C. (2021). TiO_2_ nanoparticle-induced Xanthan Gum Polymer for EOR: Assessing the underlying mechanisms in oil-wet carbonates. J. Pet. Sci. Eng..

[19] Wang W., Peng Y., Chen Z., Liu H., Fan J., Liu Y. (2022). Synergistic Effects of Weak Alkaline–Surfactant–Polymerand SiO_2_ Nanoparticles Flooding on Enhanced Heavy Oil Recovery. Energy Fuels.

[20] Al-Jaber H.A., Arsad A., Bandyopadhyay S., Abdurrahman M., Abdulfatah M.Y., Agi A., Yusuf S.M., Abdulmunem A.R., Tahir M., Nuhma M.J. (2023). Enhancing ASP Flooding by Using Special Combinations of Surfactants and Starch Nanoparticles. Molecules.

[21] Al-Jaber H.A., Arsad A., Tahir M., Nuhma M.J., Bandyopadhyay S., Abdulmunem A.R., Rahman A.F.A., Harun Z.B., Agi A. (2023). Enhancing Oil Recovery by Polymeric Flooding with Purple Yam and Cassava Nanoparticles. Molecules.

[22] Lopez-Rubio A., Flanagan B.M., Gilbert E.P., Gidley M.J. (2008). A novel approach for calculating starch crystallinity and its correlation with double helix content: A combined XRD and NMR study. Biopolymers.

[23] Suslick K.S., Didenko Y., Fang M.M., Hyeon T., Kolbeck K.J., McNamara W.B. (1999). Acoustic cavitation and its chemical consequences. Philos. Trans. R. Soc. A.

[24] Kaplan D.L. (1998). Introduction to biopolymers from renewable resources. Biopolymers from Renewable Resources.

[25] Kim H.Y., Lee J.H., Kim J.Y., Lim W.J., Lim S.T. (2012). Characterization of nanoparticles prepared by acid hydrolysis of various starches. Starch-Stärke.

[26] Angellier H., Choisnard L., Molina-Boisseau S., Ozil P., Dufresne A. (2004). Optimization of the preparation of aqueous suspensions of waxy maize starch nanocrystals using a response surface methodology. Biomacromolecules.

[27] Ramos G.A.R., Akanji L.T., Afzal W. (2020). A Novel Surfactant-Polymer/Alkaline-Surfactant-Polymer Formulation for Enhanced Oil Recovery (EOR) Processes. Energy Fuels.

[28] Cheraghian G., Nezhad S.S.K., Kamari M., Hemmati M., Masihi M., Bazgir S. (2014). Adsorption polymer on reservoir rock and role of the nanoparticles, clay and SiO_2_. Int. Nano Lett..

[29] Agi A., Junin R., Gbadamosi A., Abbas A., Azli N.B., Oseh J. (2019). Influence of nanoprecipitation on crystalline starch nanoparticle formed by ultrasonic assisted weak-acid hydrolysis of cassava starch and the rheology of their solutions. Chem. Eng. Process. Process Intensif..

[30] Nazari Moghaddam R., Bahramian A., Fakhroueian Z., Karimi A., Arya S. (2015). Comparative study of using nanoparticles for enhanced oil recovery: Wettability alteration of carbonate rocks. Energy Fuels.

[31] Viswanathan V., Laha T., Balani K., Agarwal A., Seal S. (2006). Challenges and advances in nanocomposite processing techniques. Mater. Sci. Eng. R Rep..

[32] Hu Z., Haruna M., Gao H., Nourafkan E., Wen D. (2017). Rheological Properties of Partially Hydrolyzed Polyacrylamide Seeded by Nanoparticles. Ind. Eng. Chem. Res..

[33] Tan X., Gu B., Li X., Xie C., Chen L., Zhang B. (2017). Effect of growth period on the multi-scale structure and physicochemical properties of cassava starch. Int. J. Biol. Macromol..

[34] Wang X., Wang H., Song J., Zhang Y., Zhang H. (2018). Understanding the structural characteristics, pasting and rheological behaviours of pregelatinised cassava starch. Int. J. Food Sci. Technol..

[35] Monroy Y., Rivero S., García M.A. (2018). Microstructural and techno-functional properties of cassava starch modified by ultrasound. Ultrason. Sonochem..

[36] Rahaman A., Kumari A., Zeng X.A., Adil Farooq M., Siddique R., Khalifa I., Siddeeg A., Ali M., Faisal Manzoor M. (2021). Ultrasound based modification and structural-functional analysis of corn and cassava starch. Ultrason. Sonochem..

[37] Carmona-García R., Bello-Pérez L.A., Aguirre-Cruz A., Aparicio-Saguilán A., Hernández-Torres J., Alvarez-Ramirez J. (2016). Effect of ultrasonic treatment on the morphological, physicochemical, functional, and rheological properties of starches with different granule size. Starch-Stärke.

[38] Tan W., Li Q., Wang H., Liu Y., Zhang J., Dong F., Guo Z. (2016). Synthesis, characterization, and antibacterial property of novel starch derivatives with 1,2,3-triazole. Carbohydr. Polym..

[39] Yang W., Kong X., Zheng Y., Sun W., Chen S., Liu D., Zhang H., Fang H., Tian J., Ye X. (2019). Controlled ultrasound treatments modify the morphology and physical properties of rice starch rather than the fine structure. Ultrason. Sonochem..

[40] Aminzadeh B., Chung D.H., Zhang X., Bryant S.L., Huh C., DiCarlo D.A. Influence of surface-treated nanoparticles on displacement patterns during CO_2_ injection. Proceedings of the SPE Annual Technical Conference and Exhibition.

[41] Ahmad M., Gani A., Hassan I., Huang Q., Shabbir H. (2020). Production and characterization of starch nanoparticles by mild alkali hydrolysis and ultra-sonication process. Sci. Rep..

[42] Nazarudin N., Ulyarti U., Pratama I.A., Yuwono S.D. (2023). Improving the Characteristics of Edible Film Using Modified Cassava Starch Over Ethanol Precipitation. Sci. Technol. Indones..

[43] Wang Z., Mhaske P., Farahnaky A., Kasapis S., Majzoobi M. (2022). Cassava starch: Chemical modification and its impact on functional properties and digestibility, a review. Food Hydrocoll..

[44] Fronza P., Costa A.L.R., Franca A.S., de Oliveira L.S. (2023). Extraction and Characterization of Starch from Cassava Peels. Starch-Stärke.

[45] Yekeen N., Padmanabhan E., Syed A.H., Sevoo T., Kanesen K. (2020). Synergistic influence of nanoparticles and surfactants on interfacial tension reduction, wettability alteration and stabilization of oil-in-water emulsion. J. Pet. Sci. Eng..

[46] Agi A., Junin R., Gbonhinbor J., Onyekonwu M. (2018). Natural polymer flow behaviour in porous media for enhanced oil recovery applications: A review. J. Pet. Explor. Prod. Technol..

[47] Soleimani H., Baig M.K., Yahya N., Khodapanah L., Sabet M., Demiral B.M.R., Burda M. (2018). Synthesis of ZnO nanoparticles for oil–water interfacial tension reduction in enhanced oil recovery. Appl. Phys. A Mater. Sci. Process..

[48] Kamal M.S., Adewunmi A.A., Sultan A.S., Al-Hamad M.F., Mehmood U. (2017). Recent advances in nanoparticles enhanced oil recovery: Rheology, interfacial tension, oil recovery, and wettability alteration. J. Nanomater..

[49] Kumar N., Mandal A. (2018). Surfactant Stabilized Oil-in-Water Nanoemulsion: Stability, Interfacial Tension, and Rheology Study for Enhanced Oil Recovery Application. Energy Fuels.

[50] Ku B.K., Maynard A.D. (2006). Generation and investigation of airborne silver nanoparticles with specific size and morphology by homogeneous nucleation, coagulation and sintering. J. Aerosol Sci..

[51] Campelo P.H., Sant’Ana A.S., Pedrosa Silva Clerici M.T. (2020). Starch nanoparticles: Production methods, structure, and properties for food applications. Curr. Opin. Food Sci..

[52] Le Corre D., Angellier-Coussy H. (2014). Preparation and application of starch nanoparticles for nanocomposites: A review. React. Funct. Polym..

[53] Ali J.A., Kolo K., Manshad A.K., Mohammadi A.H. (2018). Recent advances in application of nanotechnology in chemical enhanced oil recovery: Effects of nanoparticles on wettability alteration, interfacial tension reduction, and flooding. Egypt. J. Pet..

[54] Matovanni M.P.N., Distantina S., Kaavessina M. (2022). Synthesis of Cassava Starch-Grafted Polyacrylamide Hydrogel by Microwave-Assisted Method for Polymer Flooding. Indones. J. Chem..

[55] Price G.J., Smith P.F. (1993). Ultrasonic degradation of polymer solutions. III. The effect of changing solvent and solution concentration. Eur. Polym. J..

